# NSG mice humanized with allergen‐specific T‐cell lines as in vivo model of respiratory allergy

**DOI:** 10.1111/all.14263

**Published:** 2020-04-03

**Authors:** Caterina Vizzardelli, Felix Zimmann, Birgit Nagl, Claudia Kitzmüller, Ute Vollmann, Miriam Gindl, Simone Tangermann, Beatrice Jahn‐Schmid, Lukas Kenner, Barbara Bohle

**Affiliations:** ^1^ Department of Pathophysiology and Allergy Research Centre for Pathophysiology, Infectiology and Immunology Medical University Vienna Vienna Austria; ^2^ Unit of Laboratory Animal Pathology University of Veterinary Medicine Vienna Vienna Austria; ^3^ Department of Experimental and Laboratory Animal Pathology Medical University of Vienna Vienna Austria; ^4^ Ludwig Boltzmann Institute for Cancer Research (LBI‐CR) Vienna Austria

To the Editor,

Nonobese diabetic severe‐combined‐immunodeficient γc^−/−^ (NSG) mice engrafted with peripheral blood mononuclear cells (PBMC) from allergic donors develop respiratory allergy mainly mediated by allergen‐specific CD4^+^ T cells.(1) Consequently, they represent an interesting in vivo model to assess therapeutic approaches that modulate the allergen‐specific T‐cell response causing IgE‐mediated allergy. However, the frequency of allergen‐specific CD4^+^ Th2‐cells that produce high levels of IL‐4, IL‐5, and IL‐13 but low levels of IFN‐γ in the peripheral blood of allergic individuals is very low, only one of 10^3^‐10^6^ CD4^+^ T cells is specific for pollen allergens.(2, 3) Our laboratory has long‐standing expertise in the in vitro expansion of allergen‐specific CD4^+^ T‐cell lines (TCL) from allergic individuals which were successfully employed to characterize the specificity, cross‐reactivity, and MHC‐restriction of specific T cells and to evaluate different cell types for their ability to present allergen.(4, 5) Here, we assessed whether allergen‐specific TCL are applicable in the NSG mouse model of respiratory allergy.

Bet v 1‐specific T cells were expanded from PBMC of seven birch pollen (BP)‐allergic donors as described in the Supporting information 1. At day 21 of in vitro culture, TCL consisted mostly of CD3^+^CD4^+^ cells (Figure S1A) of which approximately 50% proliferated in response to recombinant (r) Bet v 1 (mean value: 56%, range 41%‐61%; Figure S1B) and to BP extract containing natural Bet v 1 but not to the negative control Bos d 5 (Figure S1C). Intracellular cytokine staining demonstrated that the majority of rBet v 1‐reactive CFSE^low^ T cells produced IL‐4, IL‐5, and IL‐13 (Figure S1D‐F). In six tested TCL, on average less than 15% of IL‐4^+^ T cells concomitantly synthesized IFN‐γ and similar results were found for IL‐5^+^ and IL‐13^+^ T cells (Figure S1G). The majority of IL‐4^+^ cells also produced IL‐5 and IL‐13 and more than 35% of T cells producing one of the Th2 cytokines concomitantly produced TNF‐α. Accordingly, supernatants from allergen‐stimulated TCL contained significantly more IL‐5 than IFN‐γ (*P* = .028, Wilcoxon signed‐rank test) and supernatants from 5/6 tested TCL contained TNF‐α (Table 1A). Epitope mapping confirmed that the cultures were oligoclonal as all but TCL4 and TCL5 responded to several peptides in the regions Bet v 1_4‐24_, Bet v 1_19‐33_, Bet v 1_28‐42_, Bet v 1_52‐66_, Bet v 1_64‐78_, Bet v 1_73‐90_, Bet v 1_85‐99_, Bet v 1_97‐111_, and Bet v 1_112‐129_ (Figure S1H). All but TCL5 harbored cells reactive with the immunodominant T‐cell–activating region Bet v 1_139‐159_.(6)

**Table 1:** Human cytokine levels in vitro and in vivo

	A. Cytokine levels [pg/ml] in supernatants of TCL						B. Cytokine levels [pg/ml] in murine sera					
	IL‐5		IFN‐γ		TNF‐α		IL‐5		IFN‐γ		TNF‐α	
TCL	medium	rBet v 1	medium	rBet v 1	medium	rBet v 1	PBS	allergen	PBS	allergen	PBS	allergen
1	4811	12 508	43.2	58.4	1153	1685	163	230	34.0	16.5	212	180
2	4164	7480	<8	<8	494	855	7.5	38.0	<8	37.0	<25	184
3	n.t.	n.t.	n.t.	n.t.	n.t.	n.t.	<5	<5	<8	<8	<25	35.1
4	<5	31.4	<8	<8	177	168	<5	<5	<8	<8	<25	<25
5	8133	11 822	<8	<8	<25	<25	<5	24.0	<8	<8	<25	<25
6	4840	16 100	<8	472	113	9593	<5	8.5	<8	55.5	47.6	120
7	2618	10 516	<8	<8	1440	1373	<5	75.1	<8	<8	<25	<25

Note

Cytokine levels were assessed in A. supernatants from TCL that had been incubated in medium or with rBet v 1 for 48 h; B. pooled sera from 5‐7 mice that had been engrafted with TCL and i.n. challenged with PBS or allergen; mean values of duplicates are shown; n.t. not tested.

NSG mice intraperitoneally (i.p.) received allergen‐specific TCL plus autologous CD3‐depleted PBMC (CD3^‐^PBMC) as source of antigen‐presenting cells plus allergen (Figure 1A). At days 13‐15, mice were intranasally (i.n.) challenged with allergen or PBS, and human cells detected in cell suspensions from lungs at day 17 contained a median of 70.3% vs 70.8% CD4^+^ T cells, 10.8% vs 10.4% CD8^+^ T cells, and 5.2% vs 5.9% B cells, whereas monocytes and NK cells were below 0.1% in either group (Figure 1B). Similar data were found in murine spleens (data not shown). Allergen‐challenged mice showed higher percentages of basophils, eosinophils, and neutrophils in bronchoalveolar lavage fluids (BALF; Figure 1C) and significantly higher airway hyperreactivity (AHR) than PBS‐challenged animals (Figure 1D). Histopathological analysis revealed a slightly increased peribronchial inflammation in allergen‐challenged compared with PBS‐challenged animals (Figure 1E) whereas goblet cell hyperplasia was not evident in lung sections of either group (data not shown). Together, NSG mice engrafted with oligoclonal allergen‐specific Th2‐cell cultures developed respiratory allergy with AHR as pre‐eminent readout. These results confirm that allergen‐specific CD4^+^ T cells are major players in this human/mouse allergy model.

**Figure 1:**
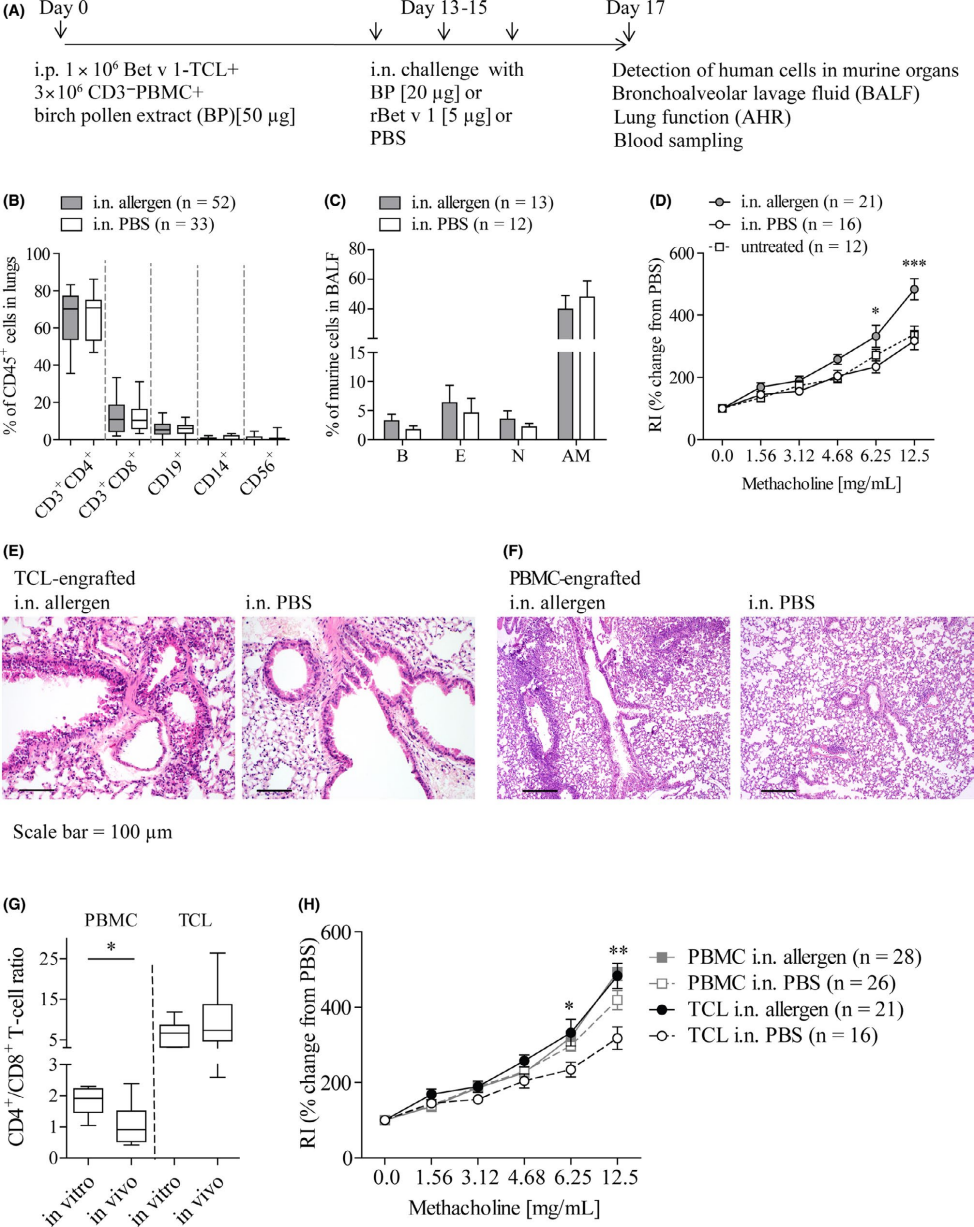
Allergic airway responses of NSG mice engrafted with allergen‐specific TCL. (A) Experimental protocol; (B) human cell types in lungs, (C) murine basophils (*B*), eosinophils (*E*), neutrophils (*N*), and alveolar macrophages (*AM*) in BALF, and (D) airway resistance (*RI*) in TCL‐engrafted mice after i.n. challenge with allergen or PBS and in untreated mice; *P < .05 between allergen‐ and PBS‐challenged mice; ***P < .001 between allergen‐, PBS‐challenged or untreated mice; (E, F) Pulmonary tissue sections stained with hematoxylin and eosin; (G) CD4^+^/CD8^+^ T‐cell ratios in PBMC and allergen‐specific TCL (in vitro) and lungs of PBMC‐ (n = 85) or TCL‐engrafted (n = 59) mice (in vivo); (H) AHR in PBMC‐ or TCL‐engrafted mice, *P < .05, **P < .01 between PBS‐challenged mice engrafted with PBMC or TCL

We observed perivascular inflammation in the lungs of PBMC‐engrafted NSG mice which is considered as an early warning of graft‐versus‐host disease (GvHD, Figure 1F).(7) Furthermore, the numbers of CD8^+^ T cells in these animals increased resulting in significant lower ratios of CD4^+^/CD8^+^ T cells in lung suspensions than in PBMC before injection (Figure 1G). No perivascular inflammation or reduced ratios of CD4^+^/CD8^+^ T cells were evident in lungs of TCL‐engrafted animals (Figure 1E,G). Moreover, mice did not show weight loss, skin rashes, ruffled fur, or increased mortality. Subclinical GvHD may cause perivascular and peribronchial inflammation and mucus production and thereby compromise allergen‐induced respiratory responses. Accordingly, PBS‐challenged PBMC‐engrafted animals showed significantly higher AHR compared with untreated mice.(7) This enhanced background was not evident in PBS‐exposed TCL‐engrafted animals (Figure 1D) and a significantly lower AHR in PBS‐exposed TCL‐engrafted than PBMC‐engrafted animals was found (Figure 1H). Allergen challenge triggered comparable AHR in both types of humanized mice. We speculate that the magnitude of allergen‐induced AHR in TCL‐engrafted mice is a consequence of an exclusive allergic response whereas it represents an overlap of allergen‐specific and GvHD‐induced inflammation in PBMC‐engrafted mice. Consequently, NSG mice engrafted with allergen‐specific TCL represent a more specific and possibly also more sensitive model of respiratory allergy.

To monitor the preservation of the human Th2 phenotype in vivo, pooled sera from 5‐7 mice i.n. challenged with allergen or PBS were analyzed for human IL‐5, IFN‐γ, and TNF‐α (Table 1B). No cytokines were detectable in untreated mice (data not shown) and mice engrafted with TCL4. Solely TNF‐α was detected in allergen‐challenged mice engrafted with TCL3. Otherwise, IL‐5 levels were higher in allergen‐challenged mice (*P* = .043, Wilcoxon signed‐rank test). Similarly, IFN‐γ and TNF‐α were predominantly found in allergen‐challenged mice. Except for mice engrafted with TCL6, IFN‐γ levels were lower than IL‐5 levels. Overall, human cytokines detected in murine sera resembled those secreted from allergen‐activated TCL in vitro (Table 1). IL‐5 is a potent activator and attractor of eosinophils and together with eotaxin‐2 induces AHR.(8) AHR, mucus production, and eosinophilia are also promoted by IL‐13 (9) which was produced by most allergen‐stimulated IL‐5^+^ T cells (Figure S1G). Like IL‐5 and IL‐13, human and murine TNF‐α demonstrate significant cross‐species reactivity. We detected no differences in the murine cytokines IL‐1β, IL‐6, and IFN‐γ in BALF and sera of allergen‐ and PBS‐exposed animals excluding a relevant role in inflammation (data not shown). It is therefore tempting to speculate that human allergen‐specific Th2 cells mediated murine airway inflammation after respiratory exposure to allergen. Together, NSG mice engrafted with allergen‐specific TCL may represent a suitable preclinical model for therapeutic approaches that modulate allergen‐specific Th2 cells.

## CONFLICT OF INTERESTS

Dr Bohle reports grants from Austrian Science Funds during the conduct of the study and personal fees from Allergen Online Database, nonfinancial support from Paul Ehrlich Institute, personal fees from Christian Doppler Research Organisation, outside the submitted work. No potential conflicts of interest were disclosed by the other authors.

## Supporting information

Supinfo
