# Supplementary material for: NSG mice humanized with allergen‐specific T‐cell lines as in vivo model of respiratory allergy
Source: Allergy. 2020 Apr 3;75(8):2081–4. doi: 10.1111/all.14263 (PMC7595002; doi:10.1111/all.14263)
Supplement: Supplementary file 1 — Supinfo [file ALL-75-2081-s001.docx]

**SUPPORTING INFORMATION**

**Supporting methods**

**Allergen-specific T-cell lines (TCL):** The study was approved by the ethics committee of the Medical University of Vienna (EK1344_2018) and all donors provided written informed consent. PBMC were stimulated with birch pollen (BP) extract (50 µg/ml, prepared as described in (1)) or rBet v 1 (5 µg/ml, Biomay, Vienna, Austria) in serum-free UltraCulture™ Medium (Lonza, Basel, Switzerland). Endotoxin levels of all proteins were below 25 EU/mg (LAL assay, Lonza). On day 5, human rIL-2 (20 U/ml Roche, Basel, Switzerland) was added. On day 7, viable T-cells were enriched by density centrifugation over Ficoll (GE Healthcare Bio-sciences, Uppsala, Sweden) and fed with rIL-2 (10 U/ml) plus irradiated (60 Gy) autologous PBMC. On day 9, rIL‐2 (10 U/ml) was added. On day 18, an aliquot of cells was incubated in duplicates with irradiated (60 Gy) autologous PBMC with/without rBet v 1 (1 µg/ml), BP extract (5 µg/ml), Bos d 5 (1 µg/ml, Sigma-Aldrich, Vienna, Austria) or 50 synthetic 12mer peptides (1 μg/ml) representing the complete amino acid sequence of Bet v 1 (2). After 48 h, [^3^H]-thymidine was added for another 16 h. Stimulation indexes (SI) were calculated as ratio of counts per minute (cpm) of TCL with stimulus and cpm of TCL without stimulus. On day 21, freshly isolated PBMC were depleted of CD3^+^ cells by incubation with Dynabeads^®^ CD3 (Invitrogen ThermoFisher Scientific) according to the manufacturer´s instruction. The resulting CD3^-^PBMC contained <2% of CD3^+^ cells. After density centrifugation TCL were analysed by flow cytometry. Another aliquot was stained with carboxyfluorescein succinimidyl ester (CFSE, Invitrogen) and cultured together with autologous CD3^-^PBMC without/with rBet v 1 (10 µg/ml) or Bos d 5 (10 µg/ml). After 4 days, proliferated cells were assessed by flow cytometry.

**Quantification of human cytokines:** Human cytokines were measured in supernatants harvested from TCL after 48 h of incubation with the indicated stimuli plus autologous CD3^-^PBMC and in murine sera using PeproTech Standard ABTS ELISA Development Kit (PeproTech, Vienna, Austria) following the manufacturer´s protocol. The limits of detection were 5 pg/ml for IL-5, 8 pg/ml for IFN-γ and 25 pg/ml for TNF-α. In sera collected from non-engrafted animals all cytokines were below the limit of detection. Unfortunately, this ELISA system did not allow the measurement of IL-13 because an unknown factor in murine sera interfered with the detection of this cytokine.

**Mouse experiments:** NSG mice were kept in the animal facilities of the Medical University of Vienna under specific pathogen-free conditions. These mice lack mature B cells, T cells, NK cells and ILCs due to the lack of the common IL-2 receptor gamma chain. All experiments were reviewed by the Institutional Review Board of the Medical University of Vienna and approved by the Ministry of Sciences (BMWF-66.009/0040-WF/V/3b/2015). 6- to 8-week-old female NSG mice received one intraperitoneal (i.p.) injection of allergen-reactive TCL (1x10^6^) together with freshly isolated autologous CD3^-^PBMC (3x10^6^) plus BP extract (50 μg). PBMC-engrafted NSG mice received one i.p. injection of PBMC (5 x 10^6^) plus BP extract (50 μg) and human rIL-4 (1000 U, Miltenyi Biotec) (4). The i.p. administration of BP extract and rIL-4 was repeated on day 7. No monitoring of the *in vivo* persistence of human cells was done. For challenges, mice were anesthetized with isofluran (Abbott, Wiesbaden, Germany) and PBS (20 µl) without or with rBet v 1 (5 μg) or BP extract (20 µg) were applied i.n.. After 48 h, invasive measurement of AHR was performed as described (4). The response to PBS was set to 100%. As no differences between the use of rBet v 1 and BP extract were observed, data were pooled. Untreated NSG mice received no TCL, CD3^-^PBMC or i.n. challenges. BALF, lung suspensions and histology were performed as described (4).

**Flow cytometry**: Human cells were stained with anti CD45-FITC, CD4-BV421 or CD4-Pe/Cy7, CD19-PE/Cy7 or CD19-APC, CD8-APC/Cy7, CD56-FITC, CD14-APC/Cy7, HLA-DR-FITC, CD80-Alexa647, CD83-PerCP (Biolegend, San Diego, CA) or CD45-eF450, CD14-PerCP, CD86-PE (eBioscience, Vienna, Austria), CD3-BV510 or simultest CD3/16^+^56^-^FITC/PE (BD Biosciences, Heidelberg Germany) as described (4). For intracellular cytokine detection, TCL were stimulated as described (3) and stained with IL-4-APC, IL-5-PE, IL-13-PerCP, TNF-α-APC/Cy7 (all from Biolegend) and IFN-γ-PE/Cy7 (eBioscience). Murine cells in BALF were stained with anti-mouse Siglec-F-BV 421 (BD Biosciences), CCR3-FITC, F4/80-BV510 (Biolegend), F4/80-FITC, CD45-PE, CD11b-PerCP/Cy5.5, CD11c-PE/Cy7, CD123-APC, GR-1-APC/eF780, and Ly-6G-APC (eBioscience).

**Statistical analysis:** Wilcoxon signed ranks test, Mann-Whitney U tests, one-way ANOVA followed by Tukey's Multiple Comparison test and two-way ANOVA followed by Bonferroni post-tests were applied to test the statistical significance of the results using the softwares SPSS10.01 (SPSS, Chicago, IL, USA) and Prism 5 (GraphPad Prism Software, Inc). All murine experiments were shown as means ± SEM; differences were considered significant if P<0.05.

**Figure S1**


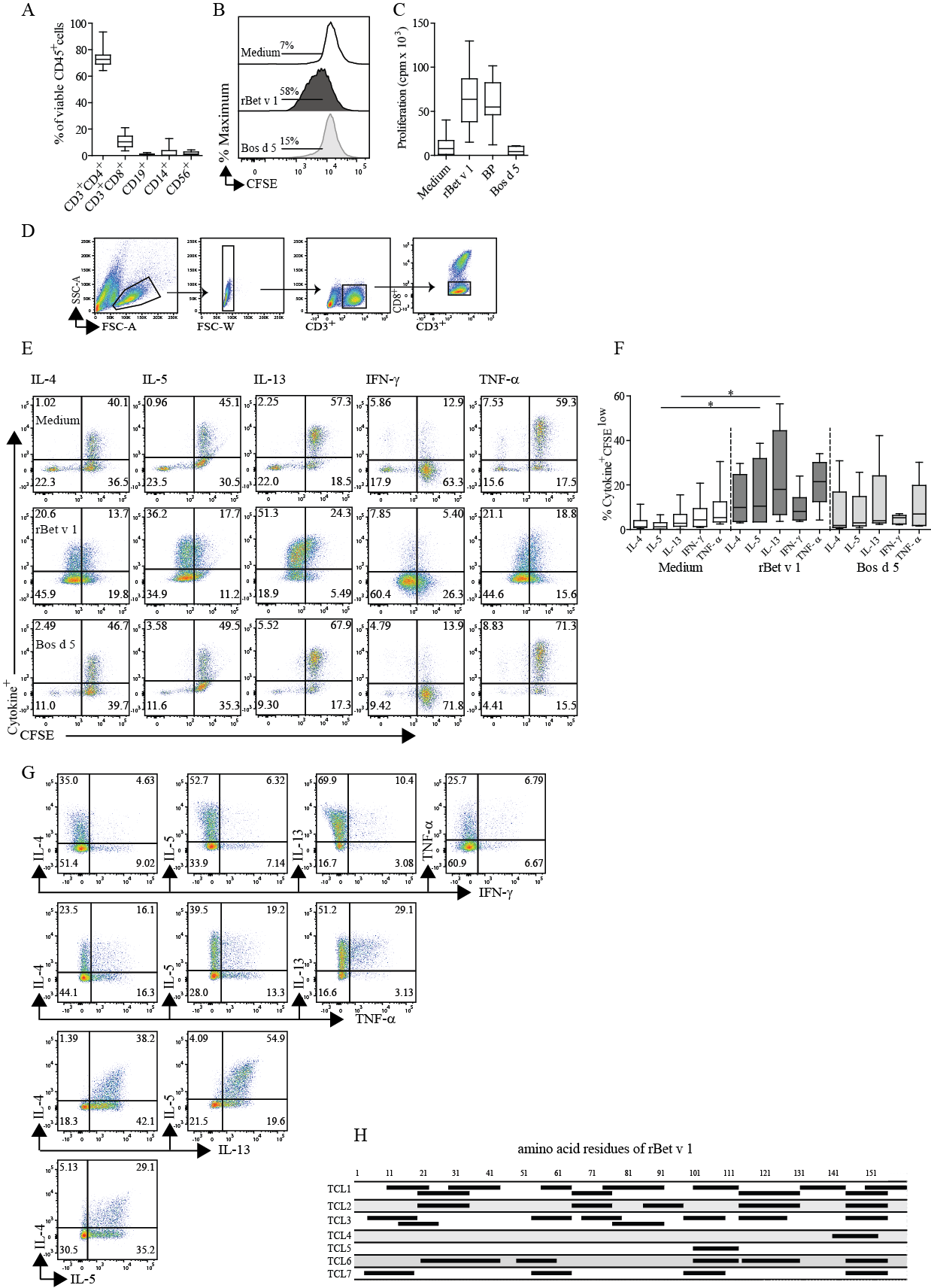


**Figure S1: Characterization of allergen-specific TCL.** (A) Cell types analysed by flow cytometry; (B) Percentage of CFSE^low^CD3^+^CD4^+^ cells in response to the different stimuli (one representative example); (C) Proliferation of TCL from 7 different BP-allergic patients to rBet v 1, birch pollen extract (BP), or Bos d 5 (cpm, counts per minute); (D) Intracellular cytokine analysis; strategy to gate CD4^+^ T-cells: viable cells were defined by SSC-A/FSC-A properties, doublets were excluded and CD4^+^ T-cells were defined as CD3^+^CD8^-^ cells; (E) Cytokine staining of CFSE-stained CD3^+^CD8^-^ cells (one representative experiment); (F) Percentages of cytokine-positive CFSE^low^CD3^+^CD8^-^ cells in 6 TCL, *P<0.05, one-way ANOVA followed by Tukey’s Multiple Comparison test; (G) Intracellular cytokine staining of Bet v 1-stimulated CFSE^low^CD3^+^CD8^-^ cells; (H) Epitope mapping: black lines depict peptides inducing SI>5.

**Acknowledgments:** This work was supported by the Austrian Science Fund project SFB F4610, Vienna, Austria. We thank Helga Schachner for preparing and staining of histological sections and Andrea Vlasaty for technical support.

**Supporting references**

1. Deifl S, Zwicker C, Vejvar E, Kitzmuller C, Gadermaier G, Nagl B, et al. Glutathione-S-transferase: a minor allergen in birch pollen due to limited release from hydrated pollen. *PLoS One* 2014;9(9):e109075.

2. Jahn-Schmid B, Radakovics A, Luttkopf D, Scheurer S, Vieths S, Ebner C, et al. Bet v 1142-156 is the dominant T-cell epitope of the major birch pollen allergen and important for cross-reactivity with Bet v 1-related food allergens. *J Allergy Clin Immunol* 2005;116(1):213-219.

3. Van Hemelen D, Mahler V, Fischer G, Fae I, Reichl-Leb V, Pickl W, et al. HLA class II peptide tetramers vs allergen-induced proliferation for identification of allergen-specific CD4 T cells. *Allergy* 2015;70(1):49-58.

4. Vizzardelli C, Gindl M, Roos S, Mobs C, Nagl B, Zimmann F, et al. Blocking antibodies induced by allergen-specific immunotherapy ameliorate allergic airway disease in a human/mouse chimeric model. *Allergy* 2018;73(4):851-861.
